# Safety and efficacy of two ilioiliac tension band plates osteosynthesis of fragility fractures of the pelvis

**DOI:** 10.1038/s41598-022-24525-7

**Published:** 2022-11-28

**Authors:** Michał Kułakowski, Paweł Reichert, Karol Elster, Paweł Ślęczka, Łukasz Oleksy, Aleksandra Królikowska

**Affiliations:** 1 Independent Public Healthcare Center in Rypin, Rypin, Poland; 2 Department of Trauma Surgery, Clinical Department of Trauma and Hand Surgery, Faculty of Medicine, Wroclaw Medical University, Borowska 213, 50-556 Wrocław, Poland; 3 Independent Public Healthcare Center in Myslenice, Myslenice, Poland; 4 Department of Physiotherapy, Faculty of Health Sciences, Jagiellonian University Medical College, Kraków, Poland; 5 Ergonomics and Biomedical Monitoring Laboratory, Department of Physiotherapy, Faculty of Health Sciences, Wroclaw Medical University, Wrocław, Poland

**Keywords:** Clinical trials, Surgery, Fracture repair

## Abstract

The study retrospectively determined the efficacy and safety of fixation of the pelvis (FFP) fragility fractures type IV using two tension band ilioiliac locking compression plates. Forty-one patients with FFP were treated in 2017–2020. 16 patients with FFP type IV, unable to walk weight-bearing, were treated by fixation using two tension band ilioiliac locking compression plates without fixing the anterior ring. Preoperatively and one year postoperatively, the functional outcome and performance were assessed using Pelvic Discomfort Index (PDI) and Timed Up and Go (TUG) test. Pre- and postoperative hemoglobin level was evaluated. Operation time and intra-and postoperative complications were documented. One year postoperatively, an X-ray was taken. The arithmetic mean (x) and standard deviations (±) of quantitative variables were calculated. T-test for dependent samples was used for pre-and postoperative results comparison. The PDI improved (p < 0.001) from x = 81.42 ± 4.04 to x = 36.19 ± 15.58. Preoperatively none of the patients was able to perform the TUG test. Postoperatively, the result exceeded x = 13.13 ± 3.99 s. The operation lasted x = 42.80 ± 8.90 min. Hemoglobin decreased (p < 0.001) from 11.63 ± 1.11 to 9.07 ± 1.21 g/dL. No complications nor fixation loosening were noted. The study support fixation using two tension band ilioiliac locking compression plates as an efficient and safe treatment of the FFP type IV.

## Introduction

Fragility fractures become more common in elderly patients, especially in developed countries. Fragility fractures of the pelvis (FFP) are an entity with increasing frequency. In Europe, it was estimated that, for example, in Germany, the incidence of osteoporotic pelvic fractures among persons over age 60 in Germany amounts to 224 per 100 000 cases per year and is still rising^1^. Approximately 10 million Americans over 50 have osteoporosis, with 34 million at risk. Osteoporotic fractures in the USA are prevalent, with an estimated 1.5 million suffering from fragility fractures yearly^2^. Treating these fractures is a big and intensifying challenge for health systems. Hip fracture cases alone are predicted to rise from 1.66 million in 1990 to an estimated 6.26 million globally by 2050^3^.

Rommens et al.^4^ examined 245 low-energy pelvic ring fractures in the elderly and presented a new classification system entitled Fragility Fractures of the Pelvis. The characteristics of these fractures are different from those of high-energy pelvic fractures in younger adults. As a result of decreased bone mineral density, typical features in the anterior and posterior pelvic rings occur. The subtypes represent different localisations of fractures and distinguish between four categories with increasing loss of stability^5^. Instability is related to pain, loss of mobility, and loss of independence. The treatment concepts of FFP remain controversial. There are older studies showing good long-term results for conservative treatments^6^. But many comparative studies show better results after operative treatment^7^. Alternatively, surgical treatment, especially for unstable fractures, allows early mobilization with better long-term results. Many authors favour surgical treatment for better pain relief, faster mobilization, and shorter recovery periods. Conservative or surgical treatment is recommended depending on the amount of stability, and instability is a strong predictor of the need for operative treatment. In the first two types of FFP, conservative treatment is advocated. In FFP type II, if conservative treatment fails, surgical intervention is performed. Rommens et al.^8^ also observed the progress of instability among patients with FFP, mostly in type II, Type III and IV are treated operatively. The rules of stabilisation are a bit different from those implemented in high-energy pelvic fractures, and the use of less invasive methods is advocated. In geriatric patients, these principles must be adapted as follows: minimally invasive surgical treatment, stable fixation, early mobilisation, and as good as possible reduction^9^. Anatomic reduction is no longer crucial. Stable fixation with a minimally invasive intervention becomes the treatment of choice. Stabilising FFP with iliosacral screws is well documented. Schmerwitz et al.^10,11^ published a series of 53 patients with FFP, treated with a single locked compression plate without fixing the anterior ring, but no literature has addressed the use of two ilioiliac plates. Hopf et al.^12^ published a series of 30 patients with osteoporotic fractures treated with from one to three iliosacral screws, also without fixing the anterior ring. Our former experience shows that using iliosacral screws is extremely difficult among osteoporotic patients. One problem is visualisation of the osteoporotic bone, and the second one is residual displacement, which makes the safe corridors for the insertion of the screws quite narrow. These problems forced us to fix these fractures with ilioiliac plates. We used to stabilise pelvic fractures with two ilioiliac plates in trauma patients, when there were contraindications of fixing the anterior ring or a bad patient’s condition made long-lasting surgery impossible, and we did not observe any hardware loosening, and we adopted this method in geriatric patients.

Faster mobilisation is especially important for the elderly patients. Various operative methods are presented in the literature with similar results and complication rates^5^. Wähnert et al.^13^ presented a new method of percutaneous iliosacral screw fixation with cement-augmented screws with good postoperative pain reduction. Kobbe et al.^14^ introduced minimally invasive stabilisation of the posterior pelvic ring with a transiliac locked compression plate with good functional results, but in their study, trauma not osteoporotic patients were operated and only four patients from 23 had only posterior ring stabilized. Good results of stabilizing FFP types III and IV with posterior locked compression plate were also observed by Schmerwitz et al.^10,11^.

Therefore, the present study aimed to retrospectively determine the efficacy and safety of fixation of the FFP type IV using two tension band ilioiliac locking compression plates without fixing the anterior ring.

## Materials and methods

The present cohort single-center retrospective study was conducted in accordance with the Declaration of Helsinki and approved by the bioethical committee of the Kujavian-Pomeranian Local Medical Chamber. All patients signed informed consent.

The initial sample consisted of 41 patients with FFP who were treated between October 2017 and December 2020 at the Independent Public Healthcare Centre in Rypin, Poland.

As the indications for surgical treatment of FFP proposed by Rommens and Hofmann were type IV of FFP and inability to walk weight-bearing^4^, the studied sample, included patients over 65 years old diagnosed with osteoporotic FFP type IV who were unable to walk weight-bearing.

### Intervention

**Figure 1:**
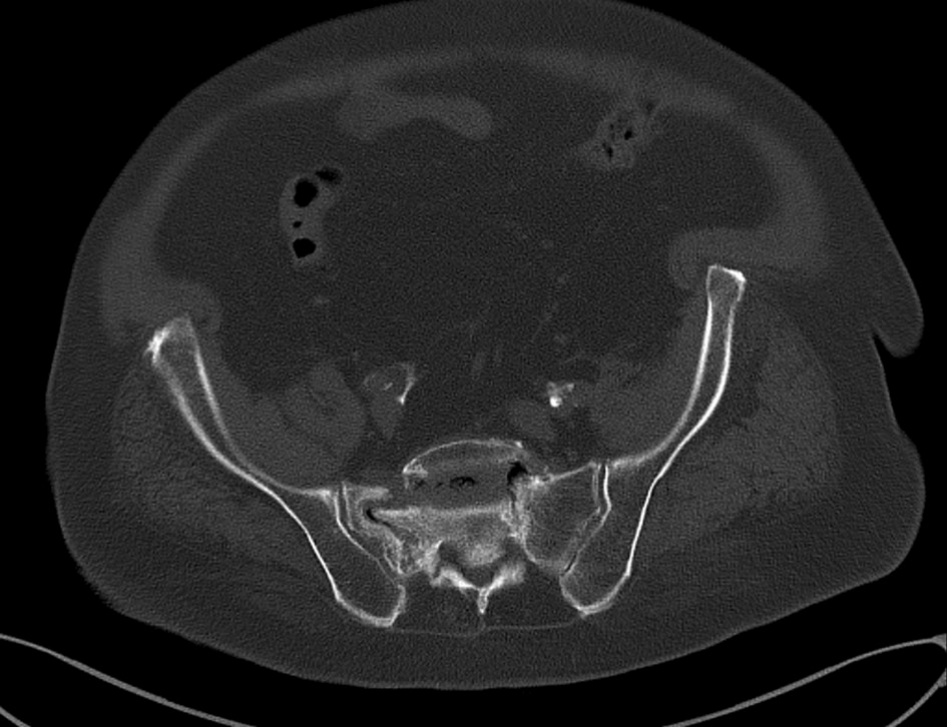
Bilateral fracture of the sacrum with moderate displacement on the right and slight displacement on the left side.

**Figure 2:**
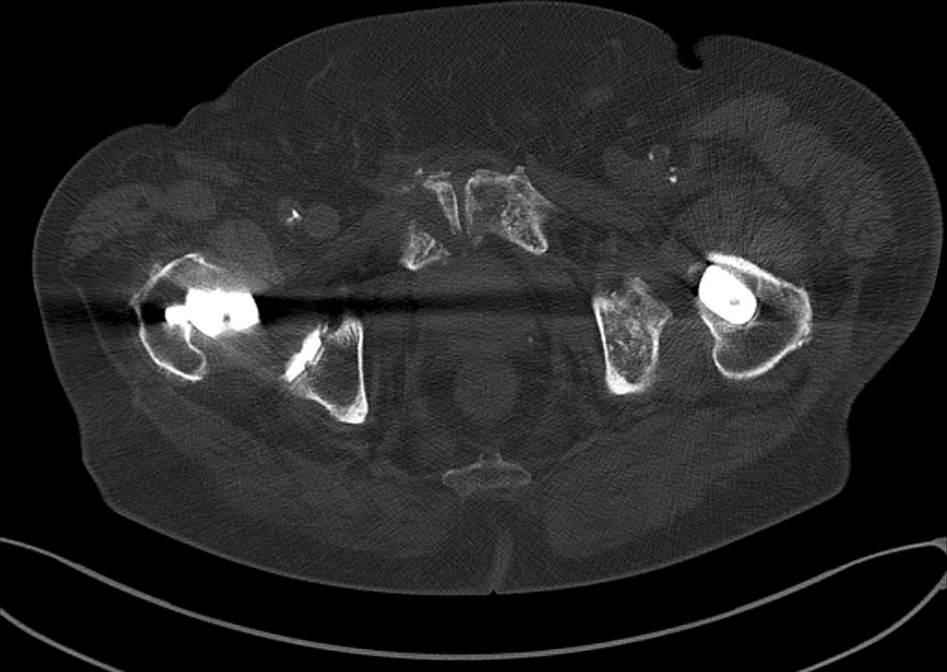
Fracture of the anterior ring showing a right-sided pubic bone fracture.

**Figure 3:**
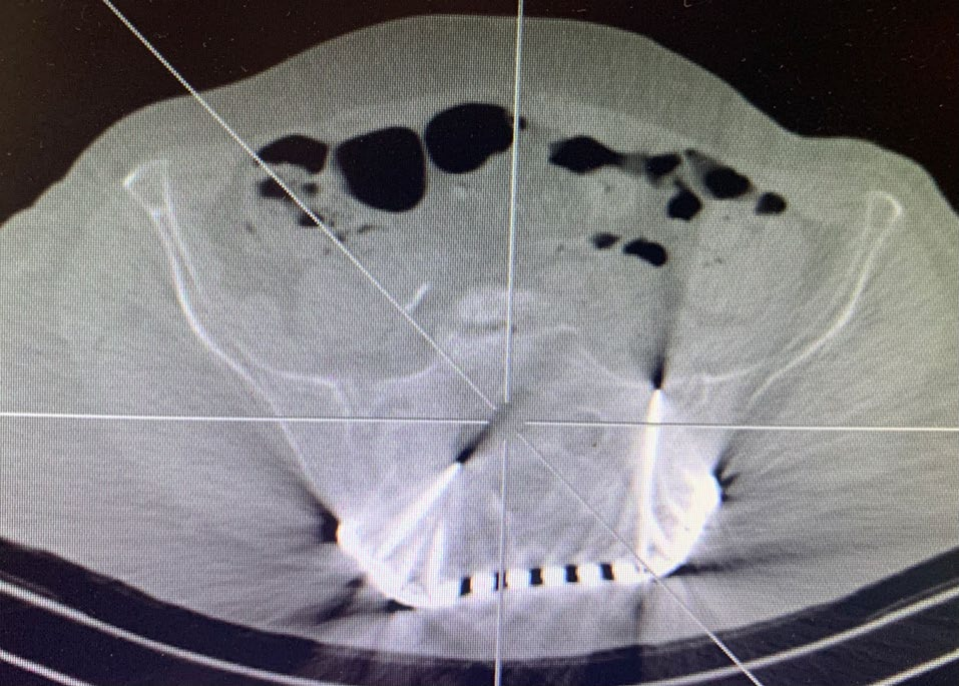
Postoperative CT of the posterior pelvis showing the sacral fractures fixed with ilioiliac plates. One screw on the right side is touching the sacral foramen.

**Figure 4:**
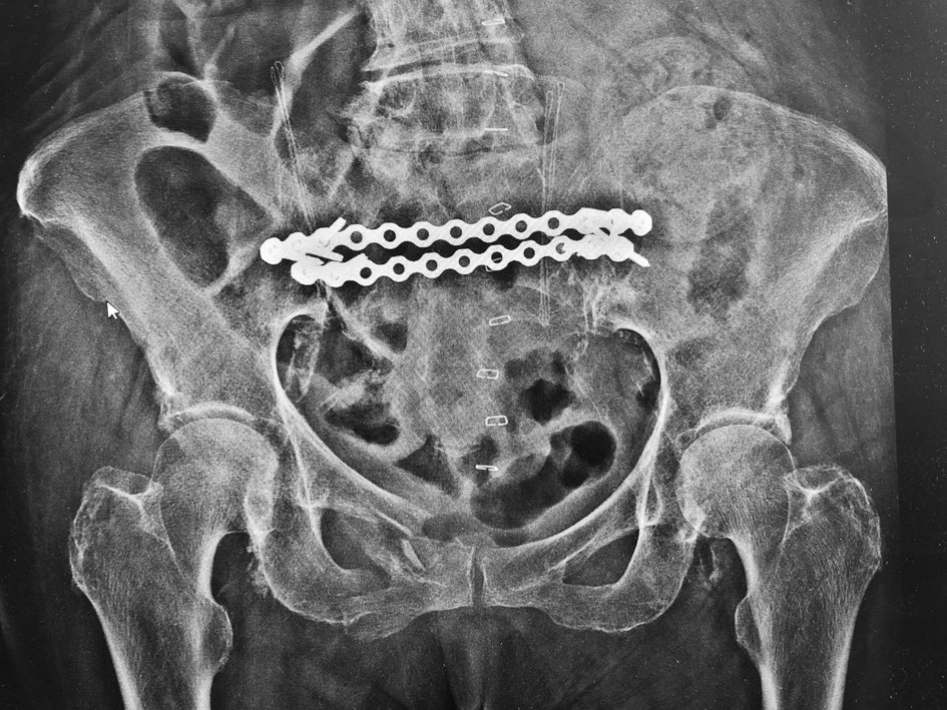
Postoperative pelvic overview showing the sacral fracture fixed with 2 ilioiliac plates.

In the operating room, the patient was in a prone position (Fig. 1). Using the C arm (Cios Fusion Siemens, Germany), the level of the sacral bone was evaluated. The plates were applied using the Krappinger modified method as presented in Figs. 2 and 3^15^. Two longitudinal incisions were made laterally from the sacroiliac joints. Next, a chisel was used to prepare a subfascial tunnel towards the opposite side, and grooves in the iliac spine were made. After bending, the locking compression plate was slid to the opposite side and after turning 180° fixed with 3.5 mm screws. The first screw was applied in a sacral bone just medially to the iliosacral joint in order to fix the plate to the bone. These screws were approximately 3–4 cm long and oriented in a sagittal axis. They were supposed to run laterally to the sacral foramina. The additional 2–3 screws on each side were inserted in the ilium, laterally to the iliosacral joint. These screws were applied about 30 degrees to the sagittal plane not to penetrate the sacral foramina and were 2–3 cm long. Two ilioiliac plates (3.5 mm system DePuy Synthes) were applied as presented in Fig. 4. The first was at the level of S1, and the second was at the level of S3. The anterior ring was not stabilised so as not to exceed the operation time and not to turn the patient over. According to former literature, we assumed fixing the posterior ring with two plates to be stable enough^10–12,16^.

### Methodology

Preoperatively and one year postoperatively, the functional outcome using Pelvic Discomfort Index (PDI)^17^ and functional performance using the Timed Up and Go (TUG) test^18^ were assessed. The developed by Borg et al., PDI consists of six items, including pain, walking, mobility of the hips, loss of sensation in the legs, sexual life, and operation scar. The PDI provides valid, specific, and relevant information to assess the outcome following fixation for pelvic ring injury^17^. Upon completion of the six-item questionnaire, the pelvic discomfort index was calculated. This index ranges from 0% pelvic discomfort (best) to 100% pelvic discomfort (worst). The calculation was done by adding the score for each question (between 0 points for no discomfort and 5 points for very severe discomfort) into a total score. This is then divided by the maximum potential score, which is 30 if all questions are answered, yielding the index. The suggested categorisation of discomfort is minimal (0–20%), moderate (21–40%), severe (41–60%), very severe (61–80%), and extremely severe (> 80%)^17^.

Preoperatively and 24 h postoperatively, the haemoglobin level was evaluated. The surgery duration time was measured. The occurrence of any intra-operative complications or complications within the first-year post-surgery was documented. One year postoperatively, an X-ray was taken and evaluated by determining any signs of hardware loosening.

### Statistical analysis

SPSS Statistics 28 (IBM® SPSS® Statistics, Armonk, NY, USA) and Microsoft Excel 365 (Microsoft Corporation, Redmond, WA, USA) were used for statistical analysis. The arithmetic mean (x) and standard deviations (±) of quantitative variables, including age (years) and PDI (points), TUG test result (s), haemoglobin level (g/dL), and time of surgery (minutes), were calculated. Based on a performed Shapiro–Wilk test to compare the preoperative PDI to the PDI at a one-year follow-up, the nonparametric t-test for dependent samples was used. The preoperative haemoglobin level was compared to the postoperative level using a parametric t-test for dependent samples. The statistical significance was set at p < 0.050.

## Results

The final sample consisted of 16 patients (11 females and 5 males) at a mean age of x = 77.38 ± 4.46 years. The detailed data gathered for studied patients are presented in Table 1. Preoperatively, the vast majority of patients, precisely nine, had 2 in the ASA classification. Six of the patients had 3, and one patient had 1 in the ASA classification. In the preoperative and intra-operative periods, 16 patients were assessed. However, at a one-year follow-up assessment, 15 patients took part as one of the patients had died for reasons not related to surgery.

**Table 1:** Detailed data gathered within the present study.

Patient’s number	Sex	Age (years)	Preoperatively			Time of surgery (minutes)	Postoperatively		
			ASA	HB level (g/dL)	PDI		HB level (g/dL)	PDI	TUG test (s)
1	F	72	2	12.3	80	50	11.2	46.6	12
2	F	78	3	11.8	76.6	36	9.6	43.3	9
3	M	76	2	10.7	90	40	7.8	50	13
4	F	72	1	12.2	86.6	42	10.3	43.3	7
5	F	74	2	10.3	76.6	56	8.6	53.3	15
6	F	82	3	11.7	83.3	60	8.0	56.6	13
7	F	77	2	9.8	83.3	33	7.8	16.6	10
8	F	79	2	10.2	80	39	7.2	20	8
9	M	77	2	13.6	86.6	49	10.3	50	12
10	M	81	3	11.9	80	39	8.7	16.6	14
11	F	86	3	13.3	76.6	37	10.4	20	17
12	F	85	2	10.5	83.3	42	7.6	43.3	22
13	F	79	2	11.2	80	55	9.2	16.6	17
14	M	73	3	12.8	76.6	30	10.3	46.6	12
15	F	72	2	12.1	83.3	33	9.6	20	16
16	M	75	3	11.7	80	45	8.5	n/a	n/a

ASA, American Society of Anaesthesiologists classification; HB, haemoglobin; n/a, not applicable; PDI, Pelvic Discomfort Index; TUG. Timed Up and Go.

The PDI statistically significantly (*p* < 0.001) improved from x = 81.42 ± 4.04 preoperatively to x = 36.19 ± 15.58 at a one-year follow-up. Preoperatively, none of the patients was able to perform the TUG test. At a one-year follow-up, the result of the TUG test exceeded x = 13.13 ± 3.99 s.

The surgery lasted for x = 42.80 ± 8.90 min. Haemoglobin statistically significantly decreased (p < 0.001) from 11.63 ± 1.11 to 9.07 ± 1.21 g/dL. No intra-operative or postoperative complications were noted. Also, based on the performed one-year postoperatively X-rays, no fixation loosening was determined.

## Discussion

The objective of this study was to evaluate the safety and efficacy of the treatment of FFP with two ilioiliac tensionband plates without fixing the anterior ring. Our results show that this method is safe and efficient.

Osteoporotic posterior pelvic ring fractures are common injuries in the elderly. The incidence rate rises dramatically with increasing age^4^. Most of these patients are women, which is similar to our study^19^. Patients younger than 65 were not included in our study. Ježek and Džupa assumed that these two groups of patients could not be compared concerning the energy of trauma, rate of osteoporosis, treatment options, and outcome^20^.

The aim of this study was to determine the functional outcome one year after operative treatment of FFP, where only the posterior ring was stabilised with two ilioiliac plates. Liuzza et al.^21^ compared ilioliliac plating with iliosacral screwing, showing similar results, but they observed earlier charging in the plate group.

Multiple techniques were introduced for the fixation of osteoporotic posterior pelvic fractures^22^. These are iliosacral screw fixation, transsacral bar osteosynthesis, bridge plating, and lumbopelvic fixation. Hopf et al.^12^ published a series of 30 patients with osteoporotic fractures treated with 2 iliosacral screws, without fixing the anterior ring. In their study, the pain level was highly decreased. We found inserting iliosacral screws to be a highly demanding method, especially in osteoporotic bone. Bone density and bowel gas made visualisation very difficult. Open posterior plating is a method where advanced visualisation methods are not necessary, and the difficulty of the method is not demanding. A study by Eckardt et al., showed a 20% reoperation rate after fixing FFP with SI joint screws, but they observed promising results when using trans-sacral screws. They also observed functional improvement in the TUG test, which is similar to our study^23^. Okazaki et al.^24^ presented stabilizing only the posterior ring with iliac intramedullary stabilization (ILIS) in Type IIIA FFPs. In their study, patients were allowed full weight-bearing on the first postoperative day, and they did not observe secondary hardware displacement, which is similar to our study. They also assessed that the insertion of two screws reduces the possibility of postoperative rotation and displacement of bone fragments, we also assessed that using two plates reduces mentioned above forces, and we did not observe secondary hardware displacement.

Our study showed that fixing the posterior ring with two ilioiliac plates without fixing the anterior ring is a safe surgical method and supports enough stability. This result is similar to results presented by Grüneweller et al.^25^, but they reported using newly designed hardware. The functional results of our study show improvement after one year of follow-up, although potential bias still exists in a retrospectively designed study.

Minimal blood loss and lack of intra- and postoperative complications are like those reported by Hopf et al.^12^ and Kobbe et al.^14^. Although Hopf et al., reported using SI joint screws and Kobbe et al., using one ilioiliac plate, and we used two plates with potentially bigger blood loss, we did not observe significant blood loss, forcing us to transfusion. Our results are similar to those reported by Okazaki et al.^24^, although they fixed the posterior ring with two bars and iliac screws, the extension of the surgery seems to be similar.

Kim et al.^26^ made a systematic review and meta-analysis comparing sacroiliac screw fixation versus plating for treating posterior pelvic ring fracture. They made a conclusion that sacroiliac screw fixation was superior to plate fixation in functional and radiological scores, but implant loosening was more common for the treatment of posterior ring injuries. We found it an important indicator for choosing the method of posterior ring fixation—the risk of loosening the sacroiliac screws in osteoporotic bone, and our study shows that posterior fixation with two ilioiliac plates provides enough stability. No literature has addressed the operative treatment of osteoporotic pelvic fractures with 2 ilioiliac plates. Schmerwitz et al.^10,11^ evaluated the clinical outcome of using a minimally invasive posterior locked compression plate among patients with FFP III and FFP IV and found this method to be safe with relatively low complication rates, low radiation dose, moderate operative time, and good functional outcome that is similar to our study. We also observed improvement in functional results, although nine patients still reported severe discomfort in PDI, they regained everyday activity independence. The main cause of bad results in the severe discomfort group after surgery was restricted hip mobility and residual pain during walking, but all patients presented improvement in everyday activity.

We did not perform CT scans one year after surgery, so it was difficult to assess bone union. The radiologist assessed only plain X-rays, and no hardware destabilization was observed.

Our study limitation is a retrospective study design and a small group of patients. We tried to avoid potential evaluation bias, and functional tests were evaluated by a surgeon who was not involved in operative treatment. Another prospective study comparing different methods of posterior ring fracture fixation among geriatric patients should be performed.

## Conclusion

The present study support fixation using two tension band ilioiliac locking compression plates as an efficient and safe treatment of patients with FFP type IV who are unable to walk weight-bearing. The one-year follow-up showed that the assessed fixation method improves the functional outcome and performance of treated elderly patients.

## Data Availability

All data generated during the study is contained within the article.
